# Neural Stem Cell‐Derived Extracellular Vesicles for Advanced Neural Repair

**DOI:** 10.1111/jnc.70170

**Published:** 2025-07-30

**Authors:** Eduardo H. Moretti, Ally L. Y. Lin, Luca Peruzzotti‐Jametti, Stefano Pluchino, Sabah Mozafari

**Affiliations:** ^1^ Department of Clinical Neurosciences, National Institute for Health Research (NIHR) Biomedical Research Centre University of Cambridge Cambridge UK; ^2^ Department of Metabolism, Digestion and Reproduction Imperial College London London UK

**Keywords:** extracellular vesicles (EVs), neural stem cells (NSCs), neurodegeneration, neuroinflammation, regenerative neuroimmunology

## Abstract

The limited regenerative capacity of the central nervous system (CNS) severely hinders treatment of neurodegenerative and neuroinflammatory diseases. These conditions, frequently exacerbated by aging, share common hallmarks such as neuroinflammation, demyelination, and neuronal loss. While neural stem cells (NSCs) hold great therapeutic promise due to their paracrine effects, including extracellular vesicle (EV) release, direct transplantation presents significant challenges. This review focuses on NSC‐derived EVs as a novel therapeutic strategy, as we explore their multimodal mechanisms in modulating neuroinflammation, promoting neurogenesis, and restoring cellular bioenergetics through the delivery of bioactive molecules and mitochondrial transfer. Recent advances in NSC‐EV‐based therapies for age‐associated neurodegenerative diseases are highlighted, along with key challenges in EV production, preservation, and targeted delivery. Finally, we outline future directions for translating this promising approach into effective clinical treatments.

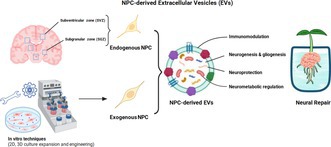

## Graphical abstract

Neurodegenerative and neuroinflammatory diseases, such as Alzheimer's and multiple sclerosis (MS), are characterized by inflammation, nerve cell damage, and a limited capacity for natural repair, which worsens with aging. While neural stem cells (NSCs) show promise for brain repair, their clinical use is limited by safety and delivery challenges. This review highlights extracellular vesicles (EVs)—tiny, bioactive particles released by NSCs—as a next‐generation, cell‐free therapy. We explore how NSC‐derived EVs can reduce inflammation, support neural regeneration and protection, and restore energy balance in damaged brain cells. This emerging strategy holds great promise for safe, targeted treatment of age‐related brain disorders.

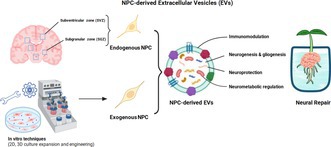

**Abbreviations**
3MA: 3‐methyladenine (autophagy inhibitor)
AD: Alzheimer's disease
ALS: amyotrophic lateral sclerosis
ANT: adenine nucleotide translocase
Areg: amphiregulin
ASIC1A: acid‐sensing ion channel subunit 1A
Asrgl1: asparaginase‐like protein 1
ATMPs: advanced therapy medicinal products
BACE1: beta‐site APP‐cleaving enzyme 1
BAG3: BCL2‐associated athanogene 3
BBB: blood–brain barrier
BDNF: brain‐derived neurotrophic factor
BEC: brain endothelial cell
CAR‐T cell: chimeric antigen receptor T cell
CD36: cluster of differentiation 36
CNS: central nervous system
COX‐2: cyclooxygenase‐2
CSF: cerebrospinal fluid
EAE: experimental autoimmune encephalomyelitis
ECM: extracellular matrix
ELOVL1: elongation of very long‐chain fatty acid protein 1
ERK: extracellular signal‐regulated kinase
ESC(s): embryonic stem cell(s)
EV(s): extracellular vesicle(s)
FGF2: fibroblast growth factor 2
FIP200: focal adhesion kinase family interacting protein of 200 kDa
GDNF: glial cell‐line‐derived neurotrophic factor
GFAP: glial fibrillary acidic protein
GPR30: G‐protein‐coupled estrogen receptor 1
HSPB8: heat shock protein beta‐8
htNSCs: hypothalamic neural stem cells
ICH: intracerebral hemorrhage
IFNβ: interferon‐beta
IL: interleukin
ILC2s: Group 2 innate lymphoid cells
iNSC(s): induced neural stem cell(s)
iPSC(s): induced pluripotent stem cell(s)
ISEV: International Society for Extracellular Vesicles
Lamp2b: lysosome‐associated membrane glycoprotein 2b
LC3B‐II: microtubule‐associated protein 1A/1B‐light chain 3B‐II
LD(s): lipid droplet(s)
LIF: leukemia inhibitory factor
LRP: low‐density lipoprotein receptor‐related protein
m6A: N6‐methyladenosine
miR‐126: microRNA‐126
miRNA: microRNA
MP: mononuclear phagocyte
MS: multiple sclerosis
MSC(s): mesenchymal stem cells
mtDNA: mitochondrial DNA
mTOR: mechanistic target of rapamycin
NGF: nerve growth factor
NOX2: NADPH oxidase 2
NSC(s): neural stem cell(s)
OPC: oligodendrocyte precursor cell
PD: Parkinson's disease
PGE2: prostaglandin E2
P‐MS: progressive multiple sclerosis
PTGS2: prostaglandin–endoperoxide synthase 2
ROS: reactive oxygen species
RVG: Rabies virus glycoprotein
SCI: spinal cord injury
SGZ: subgranular zone
siRNA: small interfering RNA
SIRT2: Sirtuin‐2
SPOP: speckle‐type POZ protein
SVZ: subventricular zone
TBI: traumatic brain injury
TLR4: toll‐like receptor 4
Tuj1: Class III beta‐tubulin
VEGF: vascular endothelial growth factor
VMB: ventral midbrain
YBX1: Y‐box binding protein 1

## 1 Introduction

Despite the presence of several stem cell‐like populations that contribute to neural regeneration, the adult human central nervous system (CNS) exhibits restricted neurogenesis (Kvistad et al. 2024), which limits its ability to repair itself effectively following aging or neurodegenerative and neuroinflammatory diseases (Adamu et al. 2024; Wheeler and Quintana 2025; Mahajan et al. 2025). These diseases encompass CNS injuries, seizure disorders (e.g., epilepsy), genetic disorders (e.g., Huntington's disease), ischemic brain injuries (e.g., stroke), cancers (e.g., glioma), neuromuscular disorders [e.g., amyotrophic lateral sclerosis (ALS)], neurodegenerative diseases [e.g., Parkinson's disease (PD) or Alzheimer's disease (AD)], and demyelinating diseases [e.g., multiple sclerosis (MS)]. These conditions share several pathological hallmarks, including pathological protein aggregation, synaptic and neuronal network dysfunction, aberrant proteostasis, cytoskeletal abnormalities, disrupted energy homeostasis, DNA and RNA defects, chronic inflammation, and neuronal cell death (Wilson III et al. 2023; Rustenhoven and Kipnis 2022). Many of these diseases exhibit aging‐associated pathological pathways or are exacerbated by the aging process (Nicaise et al. 2020; Horgusluoglu et al. 2017) making aging a primary risk factor for the majority of neurodegenerative diseases (Hou et al. 2019; López‐Otín et al. 2013; Qin et al. 2025). Additionally, aging alone leads to chronic low‐grade inflammation, termed ‘*inflammaging*’, which contributes to the worsening of neurodegenerative processes (Izquierdo 2025). A recent study reveals that aging in the mouse brain is marked by widespread upregulation of inflammatory genes and reduced synaptic function, with white matter fiber tracts—particularly in females—emerging as key sites of inflammation driven by microglial activation, astrogliosis, and myelin loss (Wang, Cui, et al. 2025). Despite significant progress in understanding the mechanisms underlying neurodegenerative and neuroinflammatory diseases, regenerative treatments remain limited.

Recent advancements in neural stem cell (NSC) research and technology have positioned them as a central focus in efforts to restore neurological function. Growing evidence shows that endogenous NSCs in mammals and humans can shift between quiescence and active proliferation, supporting neurogenesis and gliogenesis during inflammatory CNS disorders (Martino and Pluchino 2006; Okano and Sawamoto 2008; Liu et al. 2022; Ruetz et al. 2024). However, this spontaneous regeneration is insufficient to achieve full structural or functional CNS repair largely due to the complex inflammatory and inhibitory microenvironment, which becomes progressively more detrimental with aging (Zhang, Xu, et al. 2025). Aging can negatively impact this regenerative capacity, as the balance between quiescent and activated NSCs shifts significantly with increasing biological age, disrupting normal homeostatic functions and impairing the brain's ability to respond effectively to injury or disease (Obernier and Alvarez‐Buylla 2019; Nicaise et al. 2020; Ruetz et al. 2024; Bi et al. 2025; Murley et al. 2025). The maintenance of NSC niche homeostasis and its microenvironment is critically influenced by surrounding cell types such as ependymal cells (ECs), which—unlike other glial cells—possess the unique ability to uptake lipid particles from cerebrospinal fluid (CSF) via CD36 and Low‐Density Lipoprotein Receptor‐Related Protein (LRP), leading to lipid droplet (LD) accumulation under normal physiological conditions (Enos et al. 2019; Gajera et al. 2010). However, this lipid‐handling capacity becomes dysregulated during neuroinflammatory and aging conditions, including obesity and AD, potentially impairing NSC function and regenerative capacity (Zhang, Zhou, et al. 2025; Vanherle et al. 2025). With advancing age and the progressive decline in regenerative capacity, there is also an increased breakdown of proteostasis, reduced efficacy of DNA repair mechanisms, heightened vulnerability to oxidative stress, mitochondrial dysfunction, and accumulation of misfolded proteins (Lopez‐Otin et al. 2023; Zhang, Sun, et al. 2024; Maupin and Adams 2025; Lucchetti et al. 2025). Together, these factors amplify the risk of disease onset and progression in older individuals, making neurodegenerative and neuroinflammatory conditions more difficult to treat.

Recent studies on inflammatory CNS disorders suggest that dysfunction within the microenvironmental niches where NSCs reside may be responsible for their inability to achieve full restoration (Pluchino et al. 2008; Villeda et al. 2011; Andreotti et al. 2019; Nicaise et al. 2019). Diverse factors, including the uptake and release of soluble molecules—free or encapsulated by extracellular vesicles (EVs)—such as cytokines, extracellular matrix (ECM) components, growth factors, and neurotrophins, can alter the niche microenvironment, leading to imbalance and maladaptive changes in the dynamic processes that regulate NSCs behavior, including the maintenance of quiescence, activation of replication capacity, and differentiation (Andreotti et al. 2019; Martino and Pluchino 2006; Willis et al. 2022; Willis, Nicaise, Peruzzotti‐Jametti, and Pluchino 2020; Bi et al. 2025). Both resident and non‐resident components of the microenvironment, including soluble factors derived from niche constituents (e.g., neurons and glial cells), activated resident microglia, peripherally activated immune cells, and blood‐borne factors, can contribute to this dysfunction (Carpentier and Palmer 2009; Colonna and Butovsky 2017; Yousef et al. 2019; Willis et al. 2022) or affect regeneration (Hervera et al. 2018; Zhu, Xu, et al. 2025; Bernal Vicente et al. 2025). Advancements in omics technologies, single‐cell research, and neuroimaging have enabled higher‐resolution characterization of cellular heterogeneity and intercellular interactions within the CNS microenvironment (Li, Benitez, et al. 2025; Lucchetti et al. 2025; Mosharov et al. 2025; Sanborn et al. 2025). Recent advances reveal that alterations in the NSC niche can modulate developmental gene expression programs and reshape stem cell epigenetic plasticity (Shi et al. 2024; Sheehy et al. 2022; Fitzsimons et al. 2014; Kunoh et al. 2024). A recent study revealed that group 2 innate lymphoid cells (ILC2s), which accumulate within the lesion core ventricular zones following cerebral ischemia, enhance the proliferation of NSCs through the secretion of amphiregulin (Areg) (Liu et al. 2025). Mice lacking ILC2s exhibit impaired neurological function following stroke, whereas the adoptive transfer of ILC2s or Areg administration significantly improves recovery (Liu et al. 2025). These findings demonstrate how deficiencies in ILC2s and their secreted factors can disrupt the brain tissue microenvironment and hinder repair. This evidence demonstrates that aging and a dysregulated microenvironment significantly hinder the brain's regenerative capacity, making full restoration of the CNS challenging.

Various regenerative approaches have been explored to prevent disease progression and enhance repair mechanisms by targeting the CNS microenvironment. Therapies that can directly or indirectly target the niche microenvironment to restore NSC behavior and promote neuronal regeneration could have a significant impact on the treatment of inflammatory CNS disorders (Martino and Pluchino 2006; Willis, Nicaise, Peruzzotti‐Jametti, and Pluchino 2020; Willis, Nicaise, Hamel, et al. 2020; Liu et al. 2022; Willis et al. 2022). These strategies range from small‐molecule compounds targeting neuroprotective or inhibitory pathways to advanced biotherapies such as cell‐based or innovative biomaterial‐based interventions (Riessland et al. 2024; Muraro et al. 2025; Brestoff et al. 2025; Mozafari et al. 2025; Li, Zheng, et al. 2025; Wang, Xue, et al. 2025). Small‐molecule drugs often fall short in addressing the complex nature of neurodegenerative conditions, which require coordinated interactions between immune, metabolic, vascular, and nervous systems for effective regeneration. Biotherapeutic strategies to tackle these challenges can be broadly categorized into two main approaches. The first focuses on cell‐based therapies, either by enhancing endogenous repair mechanisms—such as addressing insufficient cell quantity or poor responses to pro‐regenerative cues through neurotrophic factor delivery, ECM modification, or in situ cellular reprogramming—or by using exogenous cell therapies. Among these, neural NSCs have gained significant attention due to their ability to differentiate into various neural cell types, modulate inflammation, and secrete trophic or metabolic factors that promote neural survival and plasticity (Hijal et al. 2024). Particularly, they are noteworthy for their capacity to influence the CNS microenvironment by releasing a wide range of biological signals, thereby actively modulating both local and systemic responses to injury and inflammation (Pluchino et al. 2005, 2003; Peruzzotti‐Jametti et al. 2018). Moreover, they can be derived or isolated from various sources, including embryonic, fetal, and adult CNS tissues, or generated in vitro from embryonic stem cells (ESCs) or induced pluripotent stem cells (iPSCs). They can also be directly derived from somatic cells, via conversion into stably expandable NSCs (iNSCs). NSCs isolated from developing brain tissues or derived from ESCs or iPSC sources are more accessible and exhibit greater proliferative and differentiation capabilities compared to adult NSCs (Zholudeva et al. 2021). Interestingly, a recent study found that multipotent NSCs—referred to as peripheral NSCs—can also be isolated from mouse embryonic limb, postnatal lung, tail, dorsal root ganglia, and adult lung tissues (Han et al. 2025). The therapeutic potential of NSCs from various sources has been extensively investigated for brain regeneration in a variety of experimental models of neurodegenerative and neuroinflammatory diseases or in a few clinical trials. However, despite their significant therapeutic potential, direct NSC transplantation faces considerable challenges, including low survival rates, limited engraftment, and potential immune rejection. Additionally, the hostile microenvironment in neurodegenerative diseases, characterized by chronic inflammation and gliosis, further compromises their efficacy. Other concerns include the risk of uncontrolled differentiation, tumorigenicity, and ethical issues related to stem cell sourcing.

NSCs exert many of their biological effects through their secretome, notably via EVs—nano‐ to micro‐sized, lipid bilayer‐enclosed particles that are secreted by cells and lack replication capacity (Welsh et al. 2024). EVs function as key mediators of intercellular communication by delivering diverse cargo, including lipids, proteins, RNAs, metabolites, cytokines, and organelles such as mitochondria, thereby influencing target cell behavior and maintaining tissue homeostasis (Welsh et al. 2024; Hermann et al. 2024).

EVs are broadly classified based on size, biogenesis, and composition into three main subtypes: exosomes (30–150 nm), which are formed within the endosomal pathway and released through fusion of multivesicular bodies with the plasma membrane; microvesicles (100–1000 nm), also known as ectosomes, which originate by direct outward budding of the plasma membrane; and apoptotic bodies (50–4000 nm), which are shed from cells undergoing programmed cell death and contain nuclear fragments and organelles (Welsh et al. 2024; Li, Song, et al. 2024). Among these, small EVs (sEVs)—typically < 200 nm and enriched in endosome‐related markers—are frequently studied in neurobiological contexts due to their ability to cross the blood–brain barrier (BBB), deliver neurotropic cargo, and serve as low‐immunogenic drug carriers (Li, Song, et al. 2024; Chen et al. 2025).

Beyond the canonical subtypes, additional vesicular and non‐vesicular extracellular particles have been described, reflecting the expanding complexity of intercellular communication. These include ARMMS (arrestin domain‐containing protein 1‐mediated microvesicles) involved in Notch signaling; migrasomes, which bud from retraction fibers of migrating cells and mediate tissue remodeling; and exophers, which expel damaged cellular components under stress (Wang et al. 2018; Zhang et al. 2023; Jiang et al. 2023; Chuang et al. 2024; Siddique et al. 2021). Other notable types include large oncosomes from cancer cells, telocyte‐derived EVs involved in neurovascular signaling, and non‐vesicular particles such as exomeres and supermeres—nanostructures lacking lipid bilayers but enriched in functional proteins and signaling molecules (Welsh et al. 2024; Chen et al. 2025). These emerging nanostructures are increasingly recognized for their diagnostic value in neurodegenerative diseases and their potential in CNS‐targeted therapies.

Consistent with the minimal information for studies of extracellular vesicles (MISEV) 2023 guidelines, we avoid using terms such as “*exosome*” or “*ectosome*” unless biogenesis is clearly demonstrated through rigorous methodology. Given that most current isolation approaches yield heterogeneous EV populations, we collectively refer to them as “*EVs*” throughout this review unless specified otherwise. We also emphasize the importance of standardized EV characterization, including nanoparticle size profiling, imaging, and protein marker identification, as outlined by MISEV 2023 (Welsh et al. 2024).

The unique and multifunctional ability of EVs to influence multiple biological pathways makes them particularly attractive candidates for addressing the complex pathophysiology of neurodegenerative diseases. By modulating the microenvironment of the CNS, NSC‐derived EVs have emerged as a promising cell‐free biotherapeutic strategy, capable of replicating many of the beneficial effects of NSC transplantation while circumventing challenges related to cell survival, immune rejection, and tumorigenic risk. Particularly compelling is their ability to mediate neuroimmune interactions, promote neural repair, and modulate the progression of neurodegenerative diseases. These vesicles have been shown to influence key pathophysiological processes in both neurodegenerative and neuroinflammatory conditions, including neuroprotection, immune regulation, synaptic plasticity, and tissue regeneration.

Here we will explore recent advances in biotherapeutic approaches for treating neurodegenerative diseases, with a focus on NSC‐derived EVs. It will delve into their mechanisms of action, particularly their role in modulating inflammatory pathways, neuroprotection, and neurometabolic support, while also discussing recent progress in preclinical and clinical applications. Furthermore, we will critically examine the challenges associated with the clinical translation of NSC‐derived EVs. By addressing these aspects, this review aims to highlight the therapeutic frontiers of NSC‐derived EVs in treating neurodegenerative diseases and advancing neural regeneration.

## 2 Biotherapeutic Approaches for CNS Regeneration

In recent years, several innovative biotherapeutic strategies have emerged to promote neural repair by either activating the brain's intrinsic regenerative capacity or introducing exogenous sources of support. Although significant progress has been made—using neurotrophic factors, neutralizing antibodies, gene therapy, biomaterials, and cell‐based therapies—major challenges remain that limit the clinical translation of these approaches.

This section explores both avenues: enhancing endogenous repair mechanisms and employing exogenous interventions to highlight current advances and future directions in CNS regeneration. Finally, the unique advantages of NSC‐derived EVs and their potential to overcome the limitations of existing regenerative strategies will be outlined.

### 2.1 Targeting Endogenous Neural Repair

**FIGURE 1:**
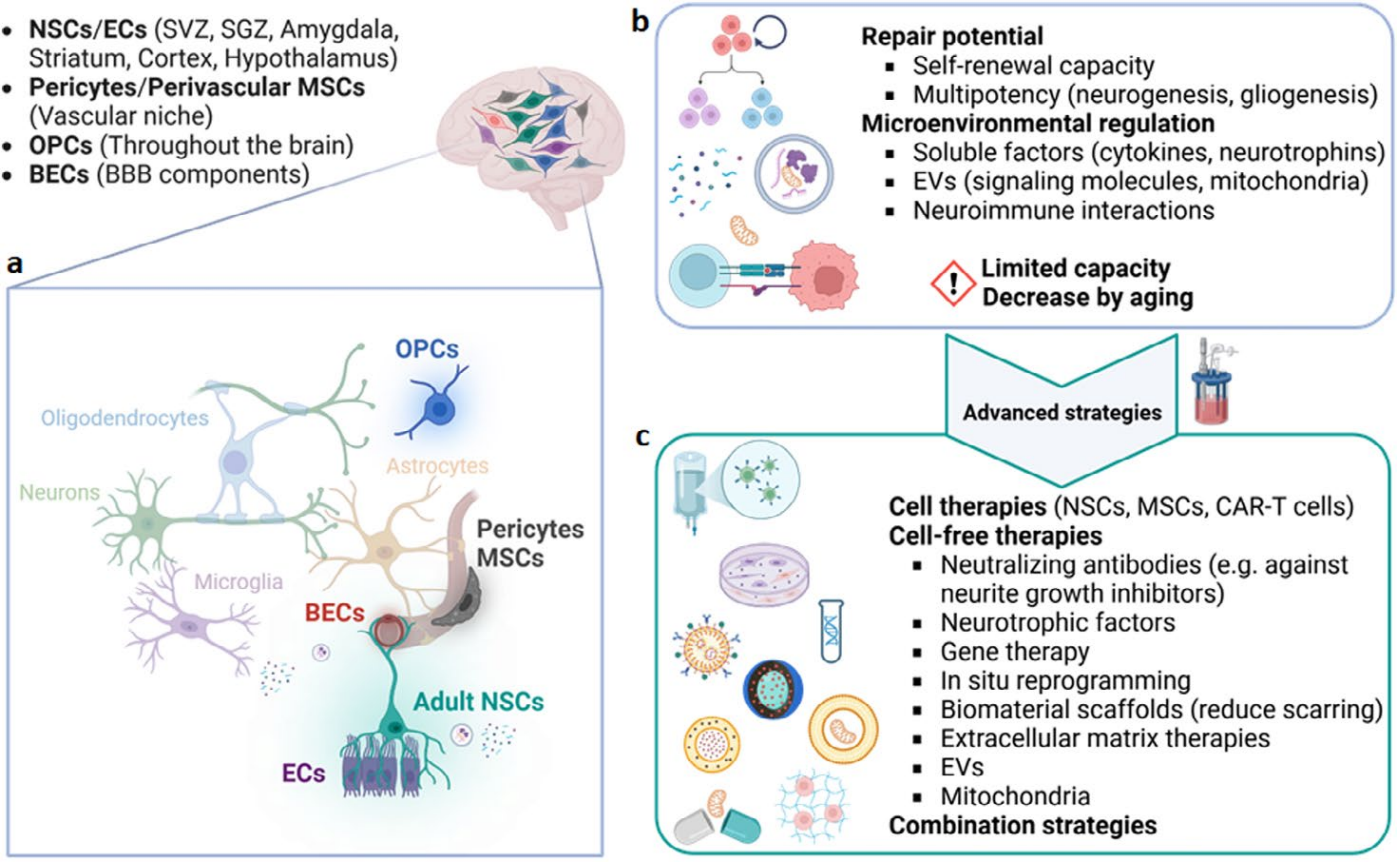
Limited endogenous repair potential and main strategies for neural regeneration. (a) Main stem cell‐like populations in the adult brain include neural stem cells (NSCs) or ependymal cells (ECs) located in neurogenic niches such as the subventricular zone (SVZ), subgranular zone (SGZ), amygdala, striatum, cortex, and hypothalamus. Oligodendrocyte precursor cells (OPCs) are distributed throughout the brain and contribute to myelination and neural repair. Perivascular mesenchymal stem cells (MSCs) and pericytes reside in the vascular niche, while brain endothelial cells (BECs) within the blood–brain barrier (BBB) exhibit progenitor‐like properties. (b) Endogenous stem cells possess self‐renewal capacity and multipotency, enabling neurogenesis and gliogenesis. Their function is tightly regulated by the microenvironment, including soluble factors (cytokines, neurotrophins), extracellular vesicles (EVs) carrying signaling molecules and mitochondria, and neuroimmune interactions. However, their repair capacity is inherently limited and declines with aging, inflammation, and metabolic dysfunctions. (c) Advanced therapeutic approaches aim to overcome the limitations of endogenous repair mechanisms. These include: *Cell‐based therapies*: NSC and MSC transplantation, engineered immune cells (e.g., CAR‐T cells); *Cell‐free therapies*: EVs, mitochondria‐based biotherapies, neutralizing antibodies (e.g., targeting neurite growth inhibitors), neurotrophic factor delivery, gene therapy, reprogramming strategies (in situ astrocyte/pericyte‐to‐neuron conversion), biomaterial and extracellular matrix‐based approaches (bio scaffolds to support neurogenesis and reduce scarring); *Combination strategies*: Integrating cell‐based and cell‐free methods for enhanced efficacy. Created by BioRender.

Experimental studies have identified key populations (Figure 1a) involved in the regenerative process in the CNS, including NSCs or ECs, which reside in neurogenic niches such as the subventricular zone (SVZ), subgranular zone (SGZ), amygdala, striatum, cortex, and hypothalamus (Jurkowski et al. 2020; Mozafari et al. 2011; Pourabdolhossein et al. 2014). In addition, oligodendrocyte precursor cells (OPCs), distributed throughout the brain, are believed to possess stem‐like capacities for myelination and neural repair (Crawford et al. 2014; Wang, Huang, et al. 2025). Perivascular mesenchymal stem cells (MSCs) and pericytes (Paul et al. 2012; Bernier et al. 2025), which reside in the vascular niche, along with brain endothelial cells (BECs) (Matsui et al. 2024) within the BBB, also exhibit progenitor‐like properties.

Among these, NSCs stand out as the most therapeutically versatile population due to several distinguishing features. They possess a tripotent differentiation capacity, allowing them to generate neurons, astrocytes, and oligodendrocytes—essential for comprehensive neural repair. NSCs are also highly responsive to environmental cues, enabling dynamic adaptation to injury or disease (Pourabdolhossein et al. 2017; Nicaise et al. 2022). Importantly, they secrete a diverse array of neurotrophic, anti‐inflammatory, and metabolic factors, supporting both cell‐autonomous and non‐cell‐autonomous mechanisms of repair (Volpe et al. 2019; Willis, Nicaise, Hamel, et al. 2020).

These features have positioned NSCs as a central focus in neuroregenerative strategies. However, endogenous NSCs in the adult human CNS are largely restricted to specific neurogenic niches and remain predominantly in a quiescent state under physiological conditions. Another major factor is the limited quantity of NSCs in endogenous pools, which may prove insufficient for addressing extensive damage, such as stroke (Bian et al. 2016). Moreover, even when present in adequate numbers, endogenous NSCs may exhibit a diminished response to pro‐regenerative cues due to inhibitory factors, inflammation, and metabolic dysregulation or age‐related decline (Pourabdolhossein et al. 2014; Tepavcevic et al. 2014; Lopez‐Otin et al. 2023).

Metabolic imbalances can disrupt nutrient‐sensing pathways crucial for NSC fate and neurogenesis (Fidaleo et al. 2017). Aging compromises the responsiveness of NSCs to regenerative signals, impairing their proliferative and differentiation capacities (Lopez‐Otin et al. 2023).

These challenges underscore the need to develop strategies that can enhance the regenerative potential of endogenous NSCs (Figure 1b).

Some advances in targeting endogenous pools include the use of neurotrophic factors, neutralizing antibodies targeting endogenous molecules within the microenvironment that inhibit CNS repair, gene therapy for cell‐target DNA repair, in situ‐directed reprogramming of CNS cells, and the development of biomaterials to modify the disrupted CNS extracellular matrix (Zamproni et al. 2021; Barker et al. 2018; Alfonsetti et al. 2023; Yuan et al. 2024; Furlan, Pluchino, Marconi, and Martino 2003; Furlan, Pluchino, and Martino 2003). Neurotrophic factors showed the potential to restore the functional integrity of dysfunctional cells, such as promoting neural or myelin regeneration in experimental models (El Ouaamari et al. 2023; Stankoff et al. 2002). The use of monoclonal antibodies targeting glial cell‐derived neurite outgrowth inhibitory factors could promote neural repair and motor recovery in spinal cord injury (SCI) patients (Weidner et al. 2025; Freund et al. 2009). However, endogenous cell‐stimulating and inhibitory neural growth factors have demonstrated limited efficacy in clinical trials, which raises concerns regarding their therapeutic viability in humans (El Ouaamari et al. 2023; Weidner et al. 2025; Freund et al. 2009). With a more target‐specific approach, gene therapy using viral and non‐viral vectors could promote DNA repair in target neurons or glial cells characterized by loss‐of‐function (LOF) or gain‐of‐function (GOF) mutations or truncations in critical proteins (Paul et al. 2022; Ling et al. 2023). The infusion of adeno‐associated virus containing an anti‐SOD1 microRNA (AAV‐miR‐SOD1) has been shown to repress the expression of the SOD1 gene in spinal cord tissue of patients with ALS (Mueller et al. 2020).

Moreover, in vivo and in situ neuronal conversion of CNS cells—such as astrocytes (Barker et al. 2018; Liang et al. 2024; Yuan et al. 2024) or pericytes (Karow et al. 2018, 2012)—allowed highly efficient and regionally tailored neuronal regeneration. Despite these advances in preclinical settings, the translation of neural cell reprogramming into clinical practice presents a considerable challenge. Finally, targeting the disrupted ECM of the CNS using bioscaffolds has emerged as a promising strategy to restore a supportive microenvironment for neural regeneration (Zamproni et al. 2021). These bioscaffolds can support neural cell anchoring, proliferation, and differentiation, while also helping to inhibit glial scar formation. By mimicking the native tissue architecture, bioscaffolds can serve as delivery platforms for stem cells, offering bioactive and physicochemical cues that enhance their survival and guide their differentiation into specific neural lineages at sites of CNS injury. However, the implantation of biomaterials in the brain—primarily aimed at treating focal degeneration—carries the risk of triggering adverse immune responses and potential rejection (Zamproni et al. 2021; Barker et al. 2018). Although most of these strategies (Figure 1c) have demonstrated potential in preclinical models, their inherent limitations—such as restricted efficacy, delivery challenges, and incomplete functional recovery—highlight the need for complementary or alternative approaches to more robustly enhance neural regeneration in the human CNS.

### 2.2 Exogenous Neural Regeneration

To enhance the limited endogenous repair capacity, various cell types have been transplanted in different neurological conditions. These primarily include NSCs or glial‐restricted progenitors derived from ESCs or iPSCs, iNSCs, MSCs, and more recently, chimeric antigen receptor T cells (CAR‐T cells) (Bonafede and Mariotti 2017; Franklin et al. 2021; Lee et al. 2016; Smith et al. 2021; Deuse and Schrepfer 2025). A recent study demonstrates that engineered iPSC‐derived microglia can serve as a CNS‐wide, pathology‐responsive delivery system for therapeutic proteins, effectively reducing Alzheimer's pathology and adapting to diverse neurological disease contexts (Chadarevian et al. 2025). While CAR‐T cell therapy has been utilized by modifying patient‐derived T cells to target CD19‐positive B cells in the brain—resetting the immune system where conventional antibodies cannot reach (Mullard 2024)—NSCs and MSCs from various sources remain among the most extensively studied cell types, demonstrating the greatest regenerative potential to date (Staff et al. 2019; Korshunova et al. 2020; Peruzzotti‐Jametti et al. 2021; Volpe et al. 2019; Pavan et al. 2025). MSCs have been derived from different tissues, including bone marrow, adipose tissue, umbilical cord, and dental pulp (Sherman et al. 2011; Rahimi Darehbagh et al. 2024; Song et al. 2018; Hernandez and Garcia 2021; Peruzzotti‐Jametti and Pluchino 2022; Ballini et al. 2017). In particular, their potent paracrine effects have been well documented (Willis, Nicaise, Hamel, et al. 2020); for example, they can shift microglia and macrophages toward an anti‐inflammatory phenotype by downregulating pro‐inflammatory cytokines such as TNF‐α and IL‐6, while upregulating anti‐inflammatory mediators like IL‐10 (Fu et al. 2023).

NSCs have garnered significant interest as potential biotherapeutics for neurodegenerative and neuroinflammatory diseases due to their expandability, ability to integrate into existing neuroglial elements, and capacity to promote functional repair (Mozafari et al. 2021, 2015; Smith et al. 2021; Zhang, Sun, et al. 2024). They support CNS regeneration by replenishing damaged or lost cells through direct differentiation while also enhancing endogenous repair via paracrine signaling, immunomodulation (Fossati et al. 2023; Pluchino et al. 2020), and glial scar regulation (Nicaise et al. 2022). Transplanted NSCs have been shown to improve neural cell survival, enhance synaptic plasticity, and stimulate endogenous regeneration in a range of neurodegenerative conditions (Lubetzki et al. 2020; Baker et al. 2017; Yang et al. 2024; Laterza et al. 2013).

Preclinical studies on myelin diseases reveal that, when administered intraparenchymally, iPSC‐derived NSCs differentiate into mature myelinating oligodendrocytes and, to a lesser extent, into astrocytes or neurons in the adult CNS (Mozafari and Baron‐Van Evercooren 2021; Mozafari et al. 2015; Wang et al. 2013; Windrem et al. 2014). While cell replacement has been considered the primary mechanism for systemically or intracerebroventricularly implanted NSCs in the diseased CNS, preclinical studies suggest that it is secondary to ‘*chaperone*’ effects, primarily involving immunomodulation and neuroprotection, which help restore homeostasis (Martino and Pluchino 2006; Pluchino et al. 2020, 2003, 2005). Their ability to secrete bioactive molecules positions them as promising candidates for promoting neural repair and regeneration (Pluchino et al. 2020; Ottoboni et al. 2020; Willis, Nicaise, Hamel, et al. 2020; Willis et al. 2022; Willis, Nicaise, Peruzzotti‐Jametti, and Pluchino 2020). They secrete neurotrophic factors like brain‐derived neurotrophic factor (BDNF), nerve growth factor (NGF), and glial cell‐line‐derived neurotrophic factor (GDNF), which support neuronal survival and growth (Actor et al. 2019). They also secrete growth factors such as vascular endothelial growth factor (VEGF) and fibroblast growth factor 2 (FGF2), which promote angiogenesis and neurogenesis (Actor et al. 2019). They can also release leukemia inhibitory factor (LIF), which stimulates endogenous brain repair (Laterza et al. 2013; Smith et al. 2021). NSCs modulate mononuclear phagocytes (MPs) through cell‐to‐cell contact and exert other immunomodulatory effects through paracrine and metabolic signaling (Pluchino et al. 2020; Rahimi Darehbagh et al. 2024; Peruzzotti‐Jametti et al. 2021). Moreover, they release EVs containing mRNAs and proteins that regulate oxidative stress, mitochondrial function, and inflammation in target cells (Manolopoulos et al. 2025; Zhu, Zhang, et al. 2025). Additionally, NSCs can transfer healthy mitochondria via EVs, restoring energy metabolism and reducing oxidative stress in target cells (Peruzzotti‐Jametti et al. 2021). Preclinical data have shown that NSCs can counteract the smoldering disease processes of progressive MS (P‐MS) and reduce the pro‐inflammatory activation of myeloid as well as astroglia cells (Pluchino et al. 2003; Peruzzotti‐Jametti et al. 2018).

At the clinical level, several trials have explored the feasibility, safety, and early efficacy of NSC transplantation for various neurological conditions, with promising results (Table 1). One of the first trials in traumatic cervical SCI (KCT0000879) showed that intraspinal transplantation of fetal‐derived NSPCs was safe and led to modest neurological improvements in 5 of 19 patients (Shin et al. 2015). In a Phase 1 trial for chronic thoracic SCI (NCT01772810), the delivery of spinal cord‐derived NSCs (NSI‐566) via intraspinal injection was found to be safe, with two of four participants showing lasting motor and sensory improvements 5 years after transplantation (Martin et al. 2024). In P‐MS, two studies demonstrated NSCs' potential to modulate disease progression. One trial (NCT03282760) administering allogeneic human NSCs intracerebroventricularly showed safety and clinical stability over 1 year in 15 patients, alongside metabolomic shifts in CSF (Leone et al. 2023). Another trial (NCT03269071) with intrathecal delivery of human fetal neural precursor cells (hfNPCs) reported favorable safety outcomes, reduced brain atrophy at higher doses, and increased levels of neuroprotective and anti‐inflammatory markers (Genchi et al. 2023). In ALS, a long‐term trial (NCT01640067) involving intraspinal transplantation of fetal hNSCs showed safety up to 60 months post‐surgery, with some transient functional improvements (Mazzini et al. 2015, 2019). A more recent trial (NCT02943850) tested neural progenitors engineered to secrete glial cell line‐derived neurotrophic factor (CNS10‐NPC‐GDNF) in ALS, demonstrating successful engraftment, GDNF expression, and no negative impact on motor function (Baloh et al. 2022). These trials highlight the growing potential of NSC‐based therapies for neurodegenerative and neurotraumatic disorders while emphasizing the need for larger, controlled studies to confirm efficacy. Other clinical trials using neural cells, including NSCs, in neurological conditions such as macular degeneration, Huntington's, Batten's, and Pelizaeus‐Merzbacher diseases have been reviewed by (Fan et al. 2023).

**TABLE 1:** Summary of clinical trials using neural stem cell transplantation in neurological disorders.

Disease (#NCT)	Clinical trial phase (duration)	Cell type/Source	Delivery method	Outcomes	Ref.
Chronic Thoracic SCI (NCT01772810)	I (5 year)	NSI‐566 (spinal cord‐derived NSCs)	Intraspinal (laminectomy + durotomy)	Safe; 2/4 showed durable motor/sensory improvement at 5 years	Martin et al. (2024)
Traumatic Cervical SCI (KCT0000879)	I/IIa (1 year)	hNSPCs (fetal telencephalon)	Intraspinal	Safe; clinical improvement in 5/19 transplanted patients	Shin et al. (2015)
P‐MS (NCT03282760)	I (1 year)	Allogeneic hNSCs	ICV	Safe; stable MRI and clinical outcomes; metabolomic changes (*n* = 15)	Leone et al. (2023)
P‐MS (NCT03269071)	I (1.8 years)	Human fetal NPCs (hfNPCs)	Intrathecal	Safe; less brain atrophy at higher dose; increased CSF biomarkers (*n* = 12)	Genchi et al. (2023)
ALS (NCT01640067)	I (1.5 years) I (2–2.5 years)	hNSCs (fetal, non‐immortalized)	Intraspinal (lumbar/cervical)	Safe up to 60 months; some motor/ALS‐FRS‐R score gains; no adverse effects (*n* = 18)	Mazzini et al. (2015, 2019)
ALS (NCT02943850)	I/IIa (1 year)	CNS10‐NPC‐GDNF (engineered NPCs)	Unilateral intraspinal (lumbar)	Safe; graft survival + GDNF expression confirmed post‐mortem (*n* = 13/18)	Baloh et al. (2022)

*Note:* This table provides an overview of key clinical trials investigating NSC transplantation across various neurological conditions, including SCI, P‐MS, and ALS.

Abbreviations: ALS, amyotrophic lateral sclerosis; ALS‐FRS‐R, ALS Functional Rating Scale–Revised; CNS10‐NPC‐GDNF, human neural progenitor cells genetically modified to express glial cell line‐derived neurotrophic factor (GDNF); CSF, cerebrospinal fluid; hfNPCs, human fetal neural progenitor cells; hNSCs, human neural stem cells; hNSPCs, human neural stem/progenitor cells; ICV, intracerebroventricular; I/IIa, clinical trial phases I and IIa; intraspinal, direct injection into the spinal cord; intrathecal, delivery into the spinal canal; KCT, Korea Clinical Trial Registry; MRI, magnetic resonance imaging; MS, multiple sclerosis; *n*, number of participants; NCT, National Clinical Trial identifier (ClinicalTrials.gov); NPCs, neural progenitor cells; NSC, neural stem cell; NSI‐566, a proprietary spinal cord‐derived human neural stem cell line; SCI, spinal cord injury; y, years.

Although NSC transplantation has shown promise in preclinical and early clinical studies by promoting neuroprotection, immunomodulation, and structural repair, its broader clinical translation remains limited by challenges such as immunogenicity, tumorigenicity, invasive delivery routes, and poor graft survival or integration. To address these limitations, increasing attention has turned toward harnessing the regenerative and immunomodulatory functions of transplanted cells through their secretome—particularly EVs. NSC‐derived EVs offer a compelling cell‐free alternative that retains many of the therapeutic effects of NSCs—such as the delivery of neurotrophic factors, immunomodulators, and even functional mitochondria—while avoiding the risks associated with cell‐based therapies. The following section details the advantages of NSC‐EV‐based biotherapy, including low immunogenicity, BBB penetration, neurotropism, and safety, combined with the functional sophistication of their parental cells, positioning them as a next‐generation regenerative platform for CNS repair.

## 3 Cell‐Free Biotherapies Based on EVs

EVs represent a potent mechanism of action in various cell transplantation studies. EVs derived from diverse cell sources have been explored for the treatment of various neurological disorders (Putthanbut et al. 2024). These include EVs derived from astrocytes, oligodendrocytes, neurons, macrophages, microglia, pericytes, brain endothelial cells, blood serum, CSF, olfactory ensheathing cells, MSCs, and NSCs. Given that each CNS disorder is defined by unique pathological mechanisms, inflammatory environments, and cellular vulnerabilities, the therapeutic application of EVs from various sources has been increasingly tailored to specific disease contexts. This has facilitated the strategic optimization of EV‐based approaches to enhance neuroprotection, modulate immune responses, and promote neuronal regeneration (Hermann et al. 2024). For instance, EVs from astrocytes, particularly in the ventral midbrain (VMB), have shown neuroprotective effects in PD models by rescuing neuronal mitochondrial function (Leggio et al. 2022). Moreover, astrocyte‐derived secretome promotes neuronal maturation and functional activity in human forebrain organoids by enhancing cortical layer development, increasing deep‐layer neuron production, and supporting resilience to cellular stress through LD accumulation (Zheng et al. 2025). Oligodendrocyte‐derived EVs contribute to axonal integrity and immune regulation in the CNS. They deliver proteins like sirtuin‐2 (SIRT2) that enhance axonal ATP production via deacetylation of mitochondrial adenine nucleotide translocase (ANT) (Fruhbeis et al. 2020; Chamberlain et al. 2021). Casella et al. reported that oligodendrocyte‐derived EVs, which naturally contain multiple myelin antigens, offer a promising antigen‐specific therapeutic strategy for autoimmune neuroinflammation like MS by restoring immune tolerance and reducing disease pathophysiology in EAE animal models, bypassing the need to identify specific target antigens (Casella et al. 2020). Oligodendrocyte‐EVs enriched with HSPB8 are taken up by microglia, promoting autophagy [LC3B‐II, BCL2‐associated athanogene 3 (BAG3)], reducing oxidative stress and ubiquitinated proteins, improving mitochondrial function, and inducing anti‐inflammatory responses (Van den Broek et al. 2022). These neurometabolic and immunomodulatory effects highlight EVs as key mediators of oligodendrocyte‐driven intercellular communication. It has been shown that in a model of traumatic brain injury (TBI), microglia‐derived EVs containing miR‐124‐3p suppress mTOR signaling, the autophagy‐associated FIP200 gene, the Rela/ApoE pathway, and the toll‐like receptor‐4 (TLR4) signaling pathway (Li et al. 2019; Yang et al. 2019; Ge et al. 2020). Moreover, endothelial cell‐EVs containing miR‐199a‐5p can reduce apoptosis by ameliorating endoplasmic reticulum stress (Yu et al. 2020). In peripheral nerve injury, pericyte‐derived EV‐mimetic nanovesicles can improve peripheral nerve regeneration in mouse models of sciatic nerve transection (Yin et al. 2022). Together, these findings underscore the therapeutic versatility of EVs from various CNS‐resident and peripheral cells, providing a foundation for exploring stem cell‐derived EVs.

Among the diverse EV sources explored, those derived from MSCs and NSCs—for some similar reasons that make these cell types attractive in transplantation—are particularly compelling for neurological therapies due to their greater accessibility and well‐established regenerative, immunomodulatory, and neuroprotective properties, rendering them more clinically translatable than EVs from many other CNS‐resident cell types (Hermann et al. 2024; Manolopoulos et al. 2025). MSC‐derived EVs particularly exhibit low immunogenicity, reducing the risk of immune response (Kou et al. 2022). MSC‐derived EVs have been shown to reduce neuroinflammation and oxidative stress, enhancing angiogenesis and promoting neurogenesis (Sankarappan and Shetty 2024; Palanisamy et al. 2023). Strategies such as preconditioning, drug loading, and surface modification have been explored to enhance their efficacy, supporting their potential as a clinically translatable approach. Engineered MSC‐derived EVs have been shown to penetrate brain microvascular endothelial cell monolayers by temporarily forming inter‐endothelial gaps and selectively targeting specific recipient cells (Yin et al. 2023). MSC‐EVs can also modulate the immune response, inducing a substantial polarization of CNS microglia to an anti‐inflammatory M2‐like state in MS models, thus improving outcomes in demyelinating conditions (Smith et al. 2021; Hermann et al. 2024) (Table 2).

**TABLE 2:** Summary of current cell‐free therapies based on EVs for neurological disorders.

EV source	Cargo	Delivery	Model	Therapeutic effects	References
Astrocytes (VM)	Mitochondrial rescue factors	In vitro	PD	Restoration of neuronal mitochondrial function; neuroprotection	Leggio et al. (2022)
Astrocytes	Protein‐ and nutrient‐enriched	Human forebrain organoids	Neurodevelopmental models	↑ cortical layer development; ↑ deep‐layer neurons; ↑ resilience to cellular stress	Zheng et al. (2025)
Oligodendrocytes	Mixed protein cargo supporting metabolic activity (not specified)	In vitro	Axonal transport (Nutrient deprivation; oxidative stress)	Restored fast axonal transport; ↓ vesicle pausing; ↑ neuronal metabolism	Fruhbeis et al. (2020)
Oligodendrocytes	SIRT2 (NAD‐dependent deacetylase)	Intracerebral injection	Sirt2 KO mouse model of axonal energy deficiency	↑ axonal ATP production; restored mitochondrial membrane potential; maintenance of axonal integrity via deacetylation of ANT1/2 mitochondrial proteins	Chamberlain et al. (2021)
Oligodendrocytes	Myelin antigens and proteins, mitochondrial and metabolic enzymes	Intravenous	MS (EAE)	Restoration of immune tolerance for myelin‐related proteins and decrease in disease severity	Casella et al. (2020)
Oligodendrocytes	Small HSPB8 and autophagy markers LC3B‐II and BAG3	In vitro (C20 microglia and primary mixed neural cells)	In vitro cellular inflammation and oxidative stress	Cellular homeostasis during chronic inflammation; ↑ formation of autophagic vesicles; ↓ mitochondrial depolarization; ubiquitinated protein levels ROS	Van den Broek et al. (2022)
Microglia	Proteins, immunomodulatory factors, signaling molecules and anti‐apoptotic regulators	In vitro (endocytosis by recipient microglia)	In vitro TNF‐α‐activated human (C20) and mouse microglia under cellular stress	↑ microglial autophagy; ↓ expression of pro‐inflammatory and apoptosis genes; ↑ cellular homeostasis and survival under stress	Van den Broek et al. (2020)
Microglia	miR‐124(‐3p)	In vitro, intravenous	Scratch‐injured HT22 neurons (in vitro), TBI	Neuroprotection; improved functional recovery; Promotion of M2 polarization of microglia; ↓ neuroinflammation; ↓ neuronal autophagy	Li et al. (2019), Yang et al. (2019), Ge et al. (2020)
Endothelial cells	miR‐199a‐5p	In vitro	Ischemic stroke	↓ apoptosis and inflammation; ↓ ER stress	Yu et al. (2020)
Pericytes	Not specified	Local injection	PNI (sciatic nerve transection)	↑ peripheral nerve regeneration; ↑ angiogenesis; functional recovery	Yin et al. (2022)
MSCs	Anti‐inflammatory and regenerative miRNAs/protein, surface‐modified EVs, drug‐loaded EVs	Intravenous, Intranasal	Multiple CNS disorders (e.g., MS, stroke, TBI, AD)	↓ neuroinflammation; ↑ Microglial M2‐like polarization; ↓ oxidative stress; ↑ neurogenesis and angiogenesis; oligodendrocyte maturation; ↑ BBB penetration; ↑ targeting	Yin et al. (2023), Kaminski et al. (2020), Losurdo et al. (2020)

*Note:* This table summarizes experimental applications of EV‐based cell‐free therapies derived from diverse cellular sources for the treatment of neurological disorders. Key information includes the source of EVs, their major functional cargo (e.g., proteins, miRNAs, mitochondria), typical delivery routes (e.g., intravenous, intranasal or in vitro), targeted disease models or clinical indications, observed therapeutic effects, and relevant references. The studies listed highlight the versatile potential of EVs in modulating neuroinflammation, supporting neuronal and axonal repair, restoring mitochondrial function, and promoting neurogenesis across a range of CNS pathologies.

Abbreviations: AD, Alzheimer's disease; ANT1/2, adenine nucleotide translocase 1 and 2; ATP, adenosine triphosphate; BAG3, Bcl‐2‐associated athanogene 3; BBB, blood–brain barrier; CNS, central nervous system; EAE, experimental autoimmune encephalomyelitis (a mouse model of multiple sclerosis); ER, endoplasmic reticulum; EVs, extracellular vesicles; HSPB8, heat shock protein beta‐8; HT22, mouse hippocampal neuronal cell line; KO, knockout; LC3B‐II, microtubule‐associated protein 1A/1B‐light chain 3B‐II (autophagy marker); miR, microRNA; MS, multiple sclerosis; MSCs, mesenchymal stem/stromal cells; NAD, nicotinamide adenine dinucleotide; PD, Parkinson's disease; PNI, peripheral nerve injury; ROS, reactive oxygen species; SIRT2, sirtuin 2 (a NAD‐dependent deacetylase); TBI, traumatic brain injury; TNF‐α, tumor necrosis factor alpha; VM, ventral midbrain.

While MSC‐derived EVs offer broad immunomodulatory and regenerative benefits, NSC‐derived EVs stand out for their specialized roles in neurodevelopment and circuit repair, making them particularly promising for neurodegenerative diseases. These EVs possess intrinsic neurogenic potential, are enriched with neurotrophic factors, and have been shown to enhance synaptic plasticity and cognitive function (Volpe et al. 2019; Li et al. 2023; Ma, Wang, et al. 2019; Spinelli et al. 2025). They support neural cell differentiation, survival, and repair, while simultaneously modulating neuroinflammation and promoting brain tissue regeneration (Willis, Nicaise, Hamel, et al. 2020; Diaz Reyes et al. 2025). Notably, unlike MSC‐EVs, NSC‐derived EVs may directly influence neurogenesis and actively contribute to the restoration of damaged neural circuits (Ottoboni et al. 2020). The following section delves into the unique therapeutic properties of NSC‐EVs and their mechanisms of action in neurodegenerative and neuroinflammatory conditions.

### 3.1 NSC‐EVs Therapy for Neurodegenerative Diseases

NSC‐derived EVs offer different therapeutic benefits of NSCs without the complexities of cell transplantation, avoiding issues like cell survival, engraftment, and differentiation. EVs derived from NSCs do not carry the same risks of uncontrolled cell proliferation, tumor formation, or improper integration associated with live cell transplants (Li et al. 2023). Moreover, these EVs are less likely to cause immune rejection compared to whole‐cell transplants, making them more suitable for broader use across different individuals. Third, the small size of NSC‐derived EVs makes them more likely to cross biological barriers, such as the BBB, thereby enhancing their delivery to neural tissues (Hermann et al. 2024; Nieland et al. 2023; Yin et al. 2023; Li et al. 2023).

NSC‐derived EVs can protect neurons through enhancing the expression of antioxidant enzymes and reducing the production of reactive oxygen species (ROS), which rescue mitochondrial dysfunction and neuronal loss in neurodegenerative diseases (Li et al. 2023). Moreover, NSC‐EVs modulate the immune response by inhibiting excessive activation of pro‐inflammatory pathways, blocking the recruitment and aggregation of peripheral immune cells such as monocytes and macrophages in acute neurological diseases, reducing pro‐inflammatory cytokines, and promoting anti‐inflammatory mediators (Li et al. 2023). NSC‐EVs can also interact with myeloid cells, altering their phenotype through the production and release of anti‐inflammatory factors such as IL‐4 and IL‐14 (Willis, Nicaise, Peruzzotti‐Jametti, and Pluchino 2020; Nicaise et al. 2022). Additionally, NSC‐derived EVs can transfer functional mitochondria, thereby modulating the pro‐inflammatory phenotype of recipient myeloid cells (Peruzzotti‐Jametti et al. 2021; Nicaise et al. 2022).

Collectively, these properties highlight NSC‐derived EVs as a promising cell‐free therapeutic approach, offering neuroprotection, immunomodulation, and regeneration while also having the potential to be used as off‐the‐shelf products, thereby circumventing the challenges associated with direct NSC transplantation.

### 3.2 Mechanisms of Action of NSC‐EVs

Accumulating evidence from preclinical animal studies suggests that NSC‐derived EVs have significant and translatable therapeutic potential, which, with further mechanistic insights, could reshape the current treatment paradigm for neurological conditions, particularly age‐associated CNS disorders. These studies have demonstrated substantial global phenotypic improvement, including immunological, physiological, and behavioral outcomes, in the animal groups treated with NSC‐derived EVs.

Moreover, preclinical findings suggest that NSC‐derived EVs mitigate key hallmarks of diseases, notably reducing neuroinflammation, lesion volume, neuronal loss, demyelination, and protein aggregation while enhancing neuroprotection, metabolic function, and synaptic activity (Madhu et al. 2024; Apodaca et al. 2021; Lee et al. 2022; Sun et al. 2019; Gao et al. 2023; Barabadi et al. 2024; Webb et al. 2018; Campero‐Romero et al. 2023).

Understanding the mechanisms of action of NSC‐derived EVs can help identify specific molecular targets, enabling their engineering and further enhancing their therapeutic efficacy and potential as biotherapeutic agents. Mechanistic studies show that NSC‐derived EVs offer multiple therapeutic advantages, including modulating neuroinflammation, promoting neurogenesis and synaptic plasticity, restoring cellular metabolism, and protecting against neurodegeneration.

#### 3.2.1 NSC‐Derived EVs as Promoter of Neurogenesis

The potential capacity of NSC‐derived EVs to promote neurogenesis and gliogenesis has fundamental roles for the replacement of cell loss associated with neurodegenerative diseases and also to switch aging‐associated quiescent NSCs to an active status and stimulate the formation of new neurons and glial cells (Ruetz et al. 2024; Murley et al. 2025). In vitro experiments using LOF and GOF approaches indicate that EVs derived from cortical‐derived NSCs regulate neurogenesis from surrounding NSCs via miR‐21a (Ma, Li, et al. 2019). These experiments find that in the LOF group, inhibition of miR‐21a led to reduced β‐tubulin expression and increased GFAP expression, indicating decreased neurogenesis and increased gliogenesis. Immunofluorescence analysis confirmed these findings, showing fewer Tuj1^+^ neurons and more GFAP^+^ astrocytes. Conversely, in the GOF group, overexpression of miR‐21a enhanced β‐tubulin expression and suppressed GFAP expression, promoting neurogenesis while inhibiting gliogenesis. These results suggest that miR‐21a plays a crucial role in NSC fate by promoting neuronal differentiation and suppressing glial differentiation (Ma, Li, et al. 2019). In another study, it was shown that iNSCs, but not cortical‐derived NSCs, abundantly secrete EVs enriched with growth factors and promote the proliferation of surrounding NSCs via extracellular signal–regulated kinase (ERK) pathways (Ma, Wang, et al. 2019). Additionally, this same ERK signaling pathway mediates the anti‐apoptotic effect of iNSC‐derived EVs and promotes neural progenitor cell survival (Ma et al. 2021). This further shows that the cellular origin of NSCs influences the composition and functional properties of their secreted EVs.

In a study using EVs derived from NSCs, the authors have shown that EVs have the potential to buffer the effect induced by H_2_O_2_ and rescue the capacity of NSCs proliferation under oxidative stress conditions (Ocana et al. 2023). In this same study, a series of immunofluorescence assays revealed that the EVs were able to promote the expression of synaptic proteins and dendritic spine development and restore the morphology of dystrophic neuron cultures in a pro‐inflammatory media (Ocana et al. 2023). In vivo, the neurogenic effects of NSC‐derived EVs are evident from their ability to increase hippocampal neurogenesis through the increased proliferation of NSCs (Upadhya et al. 2020).

**FIGURE 2:**
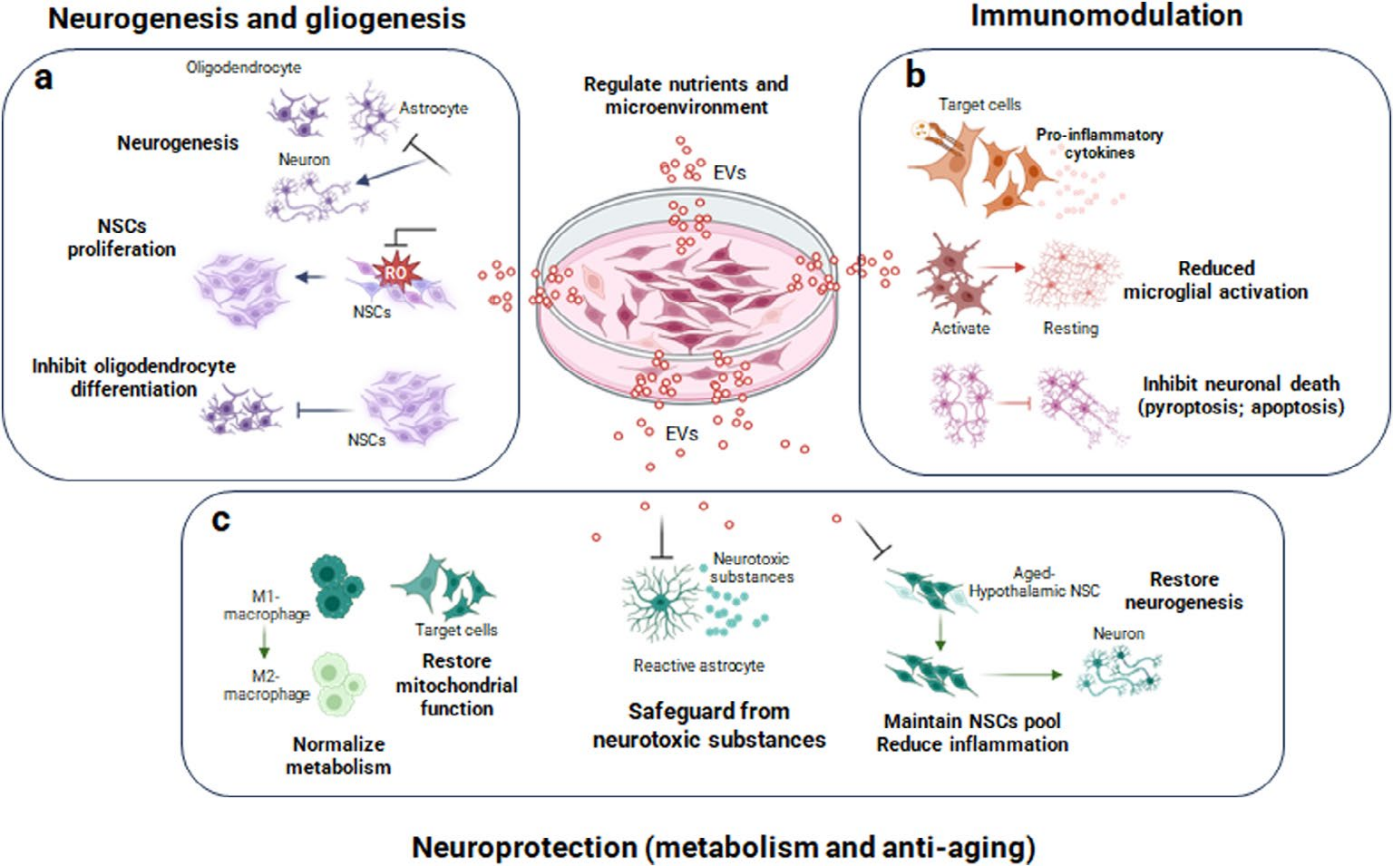
Multimodal mechanisms of action of NSC‐EVs. (a) NSC‐EVs enhance neural progenitor proliferation and differentiation through miRNA‐mediated regulation, ERK signaling, and protection against oxidative stress. They also influence oligodendrocyte differentiation via prostaglandin E2 (PGE2) signaling. (b) NSC‐EVs regulate inflammatory responses via various molecular pathways. They transfer IFN‐γ to activate Stat1 signaling in target cells and suppress microglial activation through miRNA‐mediated mechanisms. NSC‐EVs also mitigate inflammatory cell death (pyroptosis) via YBX1 and autophagy‐mediated pathways, reducing pro‐inflammatory cytokine expression. (c) NSC‐EVs protect against oxidative stress, preserve mitochondrial function by transferring intact mitochondria, regulate metabolic homeostasis, and counteract neurotoxic astrocyte activation. Additionally, NSC‐EVs suppress microglial reactivity, modulate inflammatory pathways, and mitigate aging‐associated neurodegenerative processes. Together, these multimodal actions highlight the therapeutic potential of NSC‐derived EVs in CNS repair and regeneration. Created by BioRender.

NSC‐derived EVs can also regulate oligodendrocyte differentiation after spinal cord injury via prostaglandin E2 (PGE2). Upregulating acid‐sensing ion channel 1 (ASIC1A) in NSCs raises the activity of prostaglandin‐endoperoxide synthase 2 (PTGS2), which causes EVs with high PGE2 levels to be released. These EVs act in a paracrine way and inhibit NSC differentiation into oligodendrocytes. Blocking ASIC1A or PTGS2 reduces PGE2 in EVs, reversing this inhibition and promoting oligodendrocyte differentiation (Wu et al. 2024). These studies indicate that NSC‐derived EVs foster neurogenesis and gliogenesis by influencing NSC fate, boosting neuronal differentiation, and aiding cell survival under stress, offering potential for treating neurodegenerative diseases and age‐related NSC quiescence (Figure 2a).

#### 3.2.2 NSC‐EVs as Modulators of Neuroinflammation

Current studies have demonstrated that NSC‐derived EVs are an important modulator of neuroinflammation, acting through diverse mechanisms in target cells. For example, EVs from NSCs transfer IFN‐γ via Ifngr1 to activate Stat1 signaling and induce specific activation of pro‐inflammatory cytokine signaling in fibroblast cell lines (Cossetti et al. 2014).

In microglia, EVs from NSCs seemed to exert an anti‐inflammatory role via miRNAs. Accordingly, knockdown of NSC‐derived EV‐enriched miRNAs (including let‐7i, miR‐21a, and miR‐10b) significantly reduced the inhibitory effects of EVs on Aβ‐induced microglial activation in animal models of AD (Gao et al. 2023).

EVs from NSCs can also promote anti‐inflammatory function, regulating the mechanisms of cellular death (Peng et al. 2023; Rong et al. 2019). On one hand, NSC‐derived EVs carrying Y‐box binding protein 1 (YBX1, a member of the family of DNA‐ and RNA‐binding proteins) alleviate ischemic stroke by inhibiting the process of cell pyroptosis (i.e., an inflammatory type of regulated cell death, which occurs downstream of inflammasome activation) (McKenzie et al. 2020). In this model, EVs carrying YBX1 increase m6A‐modified GPR30 stability and expression, promoting NLRP3 inflammasome ubiquitination by interacting with SPOP (speckle‐type POZ protein), ultimately suppressing neuronal pyroptosis in ischemic stroke (Peng et al. 2023). On the other hand, NSC‐derived EVs can suppress apoptosis and inflammatory processes by mediating autophagy in the SCI model in rats (Rong et al. 2019).

Moreover, EV treatment increased the expression of the autophagy marker proteins LC3B and beclin‐1 and promoted autophagosome formation in spinal neurons after SCI. This comes together with upregulated expression of the anti‐apoptotic protein Bcl‐2 and reduced expression levels of the pro‐apoptotic protein Bax, the apoptosis effector cleaved caspase‐3 (Rong et al. 2019).

Additionally, Rong et al. reported that NSC‐derived EV pretreatment inhibits microglial activation (lower number of CD68+ microglia near the injury site) and reduces neuroinflammation, exhibiting lower RNA and protein expression of pro‐inflammatory cytokines (TNF‐α, IL‐1β, and IL‐6). In the presence of the autophagy inhibitor 3‐Methyladenine (3MA), all these protective effects of EVs on spinal neurons and microglia were reversed (Rong et al. 2019), suggesting the therapeutic actions of EVs in modulating neuroinflammation and enhancing neuronal survival are at least partially mediated through autophagy.

Moreover, hiPSC‐NSC‐EVs have shown promise in mitigating Aβ‐24o‐induced neurodegeneration in vitro and in vivo in an AD mouse model by reducing neuroinflammation, amyloid plaques, and tau phosphorylation, leading to improved cognitive and mood functions (Rao et al. 2025; Madhu et al. 2024).

All in all, NSC‐derived EVs can modulate neuroinflammation and promote neuronal survival by regulating miRNAs, cell death pathways (such as pyroptosis and apoptosis), and autophagy in models of neurodegenerative diseases and injury (Figure 2b).

#### 3.2.3 NSC‐Derived EVs Provide Neuroprotection and Metabolic Support

One approach for ameliorating and delaying the progression of aging and the age‐associated neurodegenerative diseases is to promote neuroprotection, that is, an effect that may result in the salvage, recovery, or regeneration of the nervous system, its cells, structure, and function (Vajda 2002).

Most preclinical studies highlight the neuroprotective role of NSC‐derived EVs, with mechanistic studies illustrating how these EVs can mitigate the effects of aging and the hallmarks of neurodegenerative diseases. As discussed in the previous section, research by Ma et al. and Ocana et al. has already shown the neuroprotective potential of iNSC‐ and NSC‐derived EVs, particularly in oxidative stress conditions that promote cell apoptosis (Ma, Li, et al. 2019; Ma, Wang, et al. 2019; Ma et al. 2021; Ocana et al. 2023).

Another key aspect of neuroprotection is the regulation of critical nutrient concentrations and the modification of the microenvironment's physiology, which helps support neuronal health and function (Iraci et al. 2017). NSC‐derived EVs harbor L‐asparaginase activity, catalyzed by the enzyme asparaginase‐like protein 1 (Asrgl1), which has the potential of releasing aspartate that is essential for respiration and the mitochondrial electron transport chain in cell proliferation (Iraci et al. 2017; Birsoy et al. 2015; Sullivan et al. 2015). Additionally, NSCs are able to deliver functional mitochondria—with preserved membrane potential—via EVs, normalizing mitochondrial dynamics and metabolism in inflammatory immune cells, reducing pro‐inflammatory markers, and leading to clinical improvement of the Experimental Autoimmune Encephalomyelitis (EAE) animal model of MS (Peruzzotti‐Jametti et al. 2021). Transferring these mitochondria to mtDNA‐deficient cells restored mitochondrial function and improved cell survival (Peruzzotti‐Jametti et al. 2021).

Further to improve microenvironment nutrients, cellular metabolism, and stress tolerance, EVs from NSCs could safeguard cells from neurotoxic substances, such as long‐chain saturated fatty acids secreted by reactive astrocytes (Li, Zhang, et al. 2024). Reactive astrocytes become neurotoxic in mice with intracerebral hemorrhage (ICH) and in human astrocyte models, but NSC‐derived EVs can suppress this activation. Using LOF and GOF approaches, interferon‐beta (IFNβ) emerges as a key regulator of astrocyte neurotoxicity (Li, Zhang, et al. 2024). NSC‐derived EVs contain miR‐124‐3p, which degrades IFNβ mRNA and inhibits ELOVL1 expression [i.e., a metabolic enzyme that is specifically responsible for the synthesis of longer‐chain, fully saturated lipids (≥ C16:0) that are upregulated in reactive astrocytes expression; Guttenplan et al. 2021], and are able to reduce saturated lipid secretion and astrocyte neurotoxicity (Li, Zhang, et al. 2024). These mechanisms allow NSC‐derived EVs or miR‐124‐3p overexpression to mitigate neural damage, promote recovery in ICH models, and offer potential therapeutic strategies for neurological disorders by targeting neurotoxic astrocytes (Li, Zhang, et al. 2024). In another example, NSC‐derived EVs protect photoreceptors from apoptosis during retinal degeneration by inactivating reactive microglia (Bian et al. 2020). Mechanistically, the internalization of EVs by retinal microglia suppresses their activation both in vitro and in vivo, with specific miRNAs in the EVs inhibiting inflammatory signaling pathways by targeting TNF‐α, IL‐1β, and Cyclooxygenase‐2 (COX‐2) in activated microglia (Bian et al. 2020).

Another way NSC‐derived EVs promote neuroprotection is by mitigating hallmarks of the aging process, since many of these neurodegenerative conditions exhibit aging‐associated pathological pathways or are exacerbated by the aging process (Nicaise et al. 2020; Hou et al. 2019; Villeda et al. 2011). For example, hypothalamic NSCs (htNSCs) regulate aging speed partly through EVs containing miRNAs (Zhang et al. 2017). Analysis of CSF from young and middle‐aged mice revealed a decline in miRNAs produced by htNSCs with age. Inhibition of EV secretion reduced miRNA levels in the CSF without affecting cell survival or growth factor release, leading to physiological impairments in middle‐aged mice, suggesting that EV secretion from htNSCs plays a role in controlling aging. Additionally, purified EVs were shown to support htNSCs and reduce hypothalamic inflammation. In both an NSC‐ablation‐induced aging model and a normal aging model, EV treatment mitigated pro‐aging effects, such as physiological decline, without altering food intake. These findings highlight the anti‐aging properties of htNSC‐derived EVs, presenting a potential therapeutic strategy for age‐related neurodegenerative disorders (Zhang et al. 2017).

In an experimental model of brain insulin resistance (BIR)‐dependent cognitive impairment induced by insulin‐resistant NSCs, mice fed a high‐fat diet (HFD) showed reduced NSC proliferation and increased senescence of self‐renewing cells, as assessed by double‐labelling with BrdU and the immature neuron marker DCX (Natale et al. 2022). In vitro assays revealed that insulin resistance inactivated Forkhead box O1 and O3a transcription factors, inhibiting genes involved in proliferation and stemness while increasing the expression of the senescence marker p21Waf1/Cip1/Sdi1 (p21) (Natale et al. 2022). However, intranasal NSC‐derived EV treatment in HFD mice restored hippocampal neurogenesis by rebalancing proliferating and senescent NSPCs, suggesting a potential role for these EVs in preventing both physiological and pathological cognitive decline (Natale et al. 2022).

Collectively, these studies summarized in Table 3 demonstrate that NSC‐derived EVs confer neuroprotection and metabolic support by preserving mitochondrial function, regulating nutrient availability, modulating inflammatory and neurotoxic responses, and counteracting aging‐related impairments, offering promising therapeutic potential for neurodegenerative and age‐associated disorders (Figure 2c).

**TABLE 3:** Summary of recent advances in NSC‐derived EV‐based therapies in neurological conditions.

NSC source	Functional cargo	Delivery	Model/Condition	Therapeutic effects	Reference(s)
Primary mfNPCs and iNPCs from astrocytes	miR‐21a (enriched in EXOs), other miRNAs	In vitro	Differentiation of NPCs	EXOs (from primary NPCs) promoted neuronal differentiation via miR‐21a; iEXOs (from iNPCs) promoted proliferation but lacked neurogenic potential. No effect on gliogenesis noted	Ma, Li, et al. (2019)
miNPCs from fibroblasts and astrocytes	Enriched in growth factor–associated proteins; predicted activation of ERK signaling pathway	In vitro	In vitro neurogenesis/proliferation/survival assay	↑ NSC proliferation and survival; ↑ ERK activation	Ma, Wang, et al. (2019), Ma et al. (2021)
Primary mfNSCs (E13)	Catalase (antioxidant), Annexin A1, Prdx2, Txn	In vitro	In vitro PD models: (1) α‐synuclein overexpression (WT & A53T), (2) 6‐OHDA‐induced toxicity in SH‐SY5Y cells	↑ Dopaminergic neuron survival, ↓ ROS accumulation, apoptosis, and necrosis; catalase delivery confirmed	Diaz Reyes et al. (2025)
Primary mSVZ tissues from healthy 7‐ to 12‐week‐old mice	Functional mitochondria; mitochondrial proteins	ICV and in vitro	MS (EAE); mtDNA‐deficient cells	Restored mitochondrial function in recipient myeloid cells; ↓ pro‐inflammatory phenotype, improved clinical EAE score	Peruzzotti‐Jametti et al. (2021)
Primary mENPCs (SVZ, E13.5 embryos), and iNPCs	GFs (such as EGF, FGF2, and IGF2)	IV	Transient MCAO	Both NPC‐EVs and iNPC‐EVs improved sensorimotor performance and reduced neuroinflammation along with reduced apoptosis; iNPC‐EVs showed stronger anti‐apoptotic effects and enhanced cognitive recovery	Gao et al. (2022)
miNSCs from fibroblasts	miRNAs (let‐7, miR‐9, miR‐21, miR‐10b)	IV	AD model (Aβ‐induced microglial activation)	↓ Microglial and astrocyte activation; ↓ neuroinflammation; ↑ cognitive function; ↓ Aβ1‐42 deposition and phosphorylated Tau and plaque size; ↑ dendritic length/spine density; ↑ neurogenesis	Gao et al. (2023)
Primary mNPCs (SVZ‐derived, adult male mice)	Proteins associated with neuronal recovery	ICV and in vitro	Transient middle cerebral artery occlusion (tMCAO) and in vitro ischemic stroke models	No significant effect under normal neurogenesis conditions. After Ara‐C–mediated NPC proliferation inhibition, NPC‐EVs improved neurological scores and reduced infarct volume	Campero‐Romero et al. (2023)
Primary mNSCs	Not specified	In vitro	In vitro oxidative stress and inflammation‐induced neural damage	↑ Proliferation; ↑ synaptic proteins; dendritic spine restoration; rescue of dystrophic neurons	Ocana et al. (2023)
hiPSC‐NSCs	miRNAs and proteins	IN and in vitro	In vivo: Mouse model of status epilepticus In vitro: macrophage inflammatory model	Broad brain distribution after IN delivery (neurons, astrocytes, and microglia uptake); ↓ neuroinflammation; ↑ hippocampal neurogenesis, synaptic plasticity, and cognitive benefits	Upadhya et al. (2020)
mNSCs; and genetically modified (ASIC1A or PTGS2 inhibition)	PGE2‐enriched EVs regulated by ASIC1A/PTGS2 signaling	In vitro; in vivo via ASIC1A suppression	SCI model in rats and ASIC1A‐KO mice	Inhibition of oligodendrocyte differentiation by increasing PGE2‐rich EV secretion via ASIC1A activation; reversed by SIC1A/PTGS2 blockade	Wu et al. (2024)
Primary rfNSCs	Y‐box binding protein 1 (YBX1)	IV and in vitro OGD/R	Ischemic stroke (I/R injury in rats; OGD/R injury in cultured neurons)	↓ Neuronal pyroptosis via the m6A‐GPR30/SPOP/NLRP3 pathway; ↓ brain infarct volume; ↑ neuroprotection	Peng et al. (2023)
rNSCs	LC3B, beclin‐1 (autophagy‐related proteins)	IV and in vitro	Traumatic SCI in rats: in vitro models: glutamate‐induced neuronal injury and LPS‐activated microglia	Activated autophagy (↑ LC3B‐II, Beclin‐1; mitochondrial autophagosome formation); blocking autophagy (3MA) reversed these benefits; ↓ apoptosis; ↓ microglial activation and inflammation	Rong et al. (2019)
hiPSC‐NSCs	Mixed (miRNAs, proteins)	IN, in vitro	5xFAD transgenic mouse model of AD; in vitro model—Aβ‐42 oligomer‐induced neurodegeneration in human neurons	EVs incorporated into plaque‐associated microglia and astrocytes and their decreased activation; downregulation of NLRP3 inflammasome, IFN‐1, and IL‐6 signaling; Preserved phagocytosis function; ↓ Aβ plaques; ↓ tau phosphorylation; ↑ cognition and mood; ↓ neurodegeneration and oxidative stress; ↓ ROS, mitochondrial superoxide, MDA, protein carbonyls; restoration of mitochondrial membrane potential and biogenesis; regulation of apoptosis and autophagy	Rao et al. (2025), Madhu et al. (2024)
Primary hfNSCs; mNSCs	miR‐124‐3p	local at the ICH lesion site	ICH mouse model	↓ IFNβ, ↓ ELOVL1, ↓ saturated lipids; ↓ astrocyte neurotoxicity; ↓ neuronal apoptosis; motor recovery; ↓ lipid‐induced lipoapoptosis; BBB permeable. Also compared NSC‐EVs with MSC‐EVs in vitro and in vivo; NSC‐EVs demonstrated superior anti‐neurotoxic astrocyte activity	Li, Zhang, et al. (2024)
hNPCs and mNPCs	17 miRNAs (targeting TNF‐α, IL‐1β, and COX‐2); anti‐inflammatory profile	Subretinal (in vivo); in vitro microglial cultures	Retinal degeneration (rat model)	↓ retinal microglial activation; ↓ TNF‐α, IL‐1β, COX‐2; ↓ photoreceptor apoptosis; ↑ photoreceptor survival and visual function	Bian et al. (2020)
htNSCs	miRNAs (aging‐associated)	ICV	Natural aging model	↓ hypothalamic inflammation; ↑ physiological function; ↓ aging phenotypes; extended lifespan	Zhang et al. (2017)
m/aNSPCs	Not specified	IN and in vitro	Brain insulin resistance model: High‐fat diet‐induced hippocampal neurogenesis impairment; in vitro insulin‐resistant NSPCs	Restored IRS‐1/FoxO signaling; ↓ p21 expression; ↓ NSPC senescence; ↑ adult hippocampal neurogenesis; ↓ cognitive decline	Natale et al. (2022)

*Note:* This table outlines preclinical studies investigating the therapeutic potential of extracellular vesicles derived from neural stem cells (NSCs) in various neurological disease models. It includes the source and characterization of NSC‐EVs, key cargo components (e.g., miRNAs, neurotrophic factors, and proteins), delivery routes, target disease models, observed therapeutic effects, and key references. NSC‐EVs demonstrate neuroprotective, neurogenic, and immunomodulatory properties, enhancing synaptic plasticity, cognitive function, and neural repair. Unlike other cell types, NSC‐EVs are particularly suited for restoring damaged neural circuits due to their intrinsic neurodevelopmental signaling and regenerative capacity.

Abbreviations: 3MA, 3‐methyladenine; 5xFAD, transgenic mouse model carrying five familial Alzheimer's disease mutations; AD, Alzheimer's disease; ANT1/2, adenine nucleotide translocase 1 and 2; Ara‐C, cytosine arabinoside; ASIC1A, acid‐sensing ion channel subunit 1A; Aβ, amyloid‐beta peptide; BBB, blood–brain barrier; Beclin‐1, autophagy‐related protein; Catalase, antioxidant enzyme; CNS, central nervous system; COX‐2, cyclooxygenase‐2; E13, embryonic day 13; EGF, epidermal growth factor; ELOVL1, elongation of very long‐chain fatty acids protein 1; ERK, extracellular signal‐regulated kinase; EVs, extracellular vesicles; FGF2, fibroblast growth factor 2; FoxO, forkhead box O; GFs, growth factors; GPR30, G‐protein–coupled estrogen receptor 30; hfNSCs, human fetal neural stem cells; hiPSC‐NSCs, human‐induced pluripotent stem cell–derived neural stem cells; HT22, mouse hippocampal neuronal cell line; I/R, ischemia–reperfusion; IC, intracerebral; ICV, intracerebroventricular; IFN‐1/IFNβ, interferon type I/beta; IGF2, insulin‐like growth factor 2; IL‐1β, interleukin‐1 beta; IN, intranasal; IRS‐1, insulin receptor substrate 1; KO, knockout; LC3B, microtubule‐associated protein 1A/1B‐light chain 3B (autophagy marker); LPS, lipopolysaccharide; MDA, malondialdehyde; mENPCs, mouse embryonic neural progenitor cells; miNSCs, induced neural stem cells; miR, microRNA; mNPCs, mouse neural progenitor cells; mNSCs, mouse neural stem cells; mSVZ, mouse subventricular zone; mtDNA, mitochondrial DNA; NLRP3, nucleotide‐binding oligomerization domain‐like receptor protein 3; NPCs, neural progenitor cells; NSPCs, neural stem/progenitor cells; OGD/R, oxygen–glucose deprivation/reperfusion; PD, Parkinson's disease; PGE2, prostaglandin E2; Prdx2, peroxiredoxin‐2; PTGS2, prostaglandin‐endoperoxide synthase 2 (COX‐2); rfNSCs, rat fetal neural stem cells; rNSCs, rat neural stem cells; ROS, reactive oxygen species; SCI, spinal cord injury; SH‐SY5Y, human neuroblastoma cell line; SPOP, speckle‐type POZ protein; SVZ, subventricular zone; Tau, microtubule‐associated protein tau; tMCAO, transient middle cerebral artery occlusion; TNF‐α, tumor necrosis factor alpha; Txn, thioredoxin; WT, wild‐type; YBX1, Y‐box binding protein 1.

## 4 NSCs‐EVs in Clinical Settings

Encouraged by robust preclinical results, the field is now progressing toward early‐phase clinical trials using NSC‐EVs. However, the transition from bench to bedside remains far from straightforward. Despite their therapeutic promise, NSC‐EVs face substantial technological and regulatory hurdles that limit their clinical translation from bench to market. A key challenge is the large‐scale, Good Manufacturing Practice (GMP)‐compliant production of NSC‐EVs, constrained by the limited availability of high‐quality NSC sources and the absence of standardized protocols for EV isolation, purification, and characterization. Common methods such as ultracentrifugation and tangential flow filtration often fail to ensure high yield and batch‐to‐batch consistency—both essential for clinical‐grade manufacturing (Sanz‐Ros et al. 2023). Moreover, the inherent complexity and heterogeneity of EV cargo complicate the establishment of universal potency assays and quality control standards, further hindering regulatory approval. Additional concerns include the long‐term stability of stored EVs, reproducibility of delivery, and ethical considerations related to donor sourcing, particularly for fetal‐ or embryo‐derived NSCs.

To overcome these bottlenecks, advances in engineering and biomanufacturing are gaining traction. Techniques such as electroporation, transfection, and membrane fusion are being employed to enhance EV cargo loading, while surface modification with targeting peptides or antibodies improves delivery specificity. These engineering strategies, combined with scalable GMP‐compliant production systems, offer promising solutions to current limitations in yield and targeting (Ma et al. 2025). Importantly, engineered EVs preserve the biocompatibility and low immunogenicity of native vesicles while gaining programmable control for precision therapies. Although challenges in standardization and storage remain, these next‐generation hybrid biotherapeutics are rapidly emerging as a transformative platform for treating complex neurological disorders. The following sections explore the current landscape of NSC‐EV clinical trials and outline key challenges and future directions for their successful clinical implementation.

### 4.1 Clinical Trials Using NSC‐EVs

A search was performed on ClinicalTrials.gov to identify ongoing or completed studies involving NSC‐derived EVs, using “*Central Nervous System Disease*” or “*Aging/Aged*” as the condition and “*Extracellular Vesicles Derived from Neural Stem Cells*” as the intervention. The search returns 2 ongoing clinical trials investigating the potential of EVs in neurological disorders. The NouvSoma001 in Ischemic Stroke (NCT06612710) trial is assessing the safety, tolerability, and preliminary efficacy of intravenously administered iNSC‐derived EVs (NouvSoma001) for ischemic stroke treatment. Similarly, the NouvSoma001 in Neuromyelitis Optica Spectrum Disorders (NCT06620809) trial is evaluating the safety and efficacy of intrathecal administration of NouvSoma001 for neuromyelitis optica spectrum disorders. These studies, summarized in Table 4, highlight the therapeutic and diagnostic potential of NSC‐EVs in neurovascular and neuroimmune conditions.

**TABLE 4:** Ongoing clinical trials of iNSC‐derived EVs in neurological disorders.

Clinical trial	Phase (duration)	Status	Objective	Intervention	Condition
The Safety and Efficacy of NouvSoma001 in Ischemic Stroke (NCT06612710)	Phase I (up to 6 months after treatment initiation)	Ongoing	To evaluate the safety, tolerability, and preliminary efficacy of IV administration of NSC‐derived EVs	IV administration of hiNSC‐derived EVs (NouvSoma001)	Ischemic Stroke
The Safety and Efficacy of NouvSoma001 in Neuromyelitis Optica Spectrum Disorders (NCT06620809)	Phase I (up to 6 months after treatment initiation)	Ongoing	To assess the safety, tolerability, and efficacy of intrathecal administration of NSC‐derived EVs	Intrathecal administration of hiNSC‐EVs (NouvSoma001)	NMOSDs

*Note:* The table summarizes two trials evaluating the safety and efficacy of NouvSoma001 in ischemic stroke (IV route) and neuromyelitis optica (intrathecal route).

Abbreviations: EVs, extracellular vesicles; IV, intravenous; NCT, National Clinical Trial (identifier); NMOSD, neuromyelitis optica spectrum disorder; NouvSoma001, human‐induced NSC‐derived extracellular vesicles; NSC, neural stem cell; Phase I, first‐in‐human clinical trial phase focusing on safety, tolerability, and preliminary efficacy.

### 4.2 Challenges and Future Directions

The translation of the NSC‐derived EV biotherapeutic approach into clinical practice remains constrained by a range of scientific, technical, and regulatory challenges. Among these are issues related to the standardization of EV production and characterization, heterogeneity, scalability for clinical‐grade manufacturing, delivery across the BBB, and long‐term safety. Furthermore, the therapeutic efficacy of EVs is closely tied to the biological profile of their parental NSCs, making the choice of cell source a critical determinant of success.

EV characteristics are largely determined by the properties of their parent cells. Selecting an appropriate NSC source requires careful evaluation of several factors, including donor compatibility (autologous vs. allogeneic), tumorigenic factors, population purity and homogeneity, and the specific disease context (focal vs. multifocal or widespread). NSCs can be derived from various regions of the brain or spinal cord, from fetal or adult tissues, or through direct reprogramming of ES or iPS cells—each yielding EVs with distinct molecular signatures and functional capacities. Therefore, NSC source selection must be tailored to the specific neurological condition being targeted.

Historically, human NSCs isolated from fetal tissues have been a common source of EVs due to their ability to differentiate into a range of neural lineages and their therapeutic potential (Willis, Nicaise, Peruzzotti‐Jametti, and Pluchino 2020). However, the use of fetal‐derived NSCs raises significant ethical concerns, limited tissue availability, and potential immunogenicity issues, necessitating immunosuppressive therapies for recipients (Mozafari and Baron‐Van Evercooren 2021). Furthermore, fetal NSCs may also face limitations in their ability to expand in culture. ESCs, which are pluripotent and capable of differentiating into NSCs or neuroglial progenitors, are also a potential and expandable source, but they carry similar ethical concerns and the risk of immunogenicity or residual pluripotency from contaminating undifferentiated ESCs, posing a significant safety risk issue (Rahimi Darehbagh et al. 2024). The advent of iPSC technology has enabled the derivation of NSCs from adult somatic cells, circumventing their accessibility or potential immunogenicity associated with allogenic transplants (Willis, Nicaise, Peruzzotti‐Jametti, and Pluchino 2020; Rahimi Darehbagh et al. 2024). However, iPSC‐derived cells similarly carry the risks of tumorigenicity due to the potential presence of residual pluripotent cells. Additionally, some studies have identified inherent defects and altered secretomes in patient‐derived NSCs; for instance, iPSC‐derived hNSCs from patients with P‐MS, suggesting the need for rigorous health screenings before using human iPSC‐derived bioproducts in clinical settings (Willis, Nicaise, Peruzzotti‐Jametti, and Pluchino 2020). Moreover, inducing pluripotency can reset epigenetic modifications, effectively erasing age‐ or disease‐associated traits and restoring a more youthful, developmentally plastic cellular state (Cipriano et al. 2024). Another promising approach involves direct reprogramming of somatic cells, such as fibroblasts, into iNSCs, bypassing the pluripotent stage while retaining age‐associated traits from the original somatic cells, and their epigenetic modifications could be less pronounced (Wang et al. 2021). This method potentially eliminates the tumorigenicity risks associated with iPSCs and offers a more straightforward path for producing NSC‐derived products (Rahimi Darehbagh et al. 2024). However, more research is needed to assess the safety and efficacy of iNSC‐derived bioproducts, as incomplete reprogramming could lead to the presence of partially converted iNSCs in the preparations, which may compromise their safety and therapeutic potential (Nicaise et al. 2022).

Furthermore, EVs derived from NSCs of different brain regions may also vary in their therapeutic effects. For instance, EVs from a human fetal NSC line displayed neuroprotective properties against oxidative stress in vitro, while hypothalamic NSC‐derived EVs demonstrated endocrine‐like effects, influencing neurogenesis and systemic aging in mice (Bonetto and Grilli 2023). The long‐term therapeutic effects and safety profiles of NSC‐derived EVs also need further evaluation (Jin et al. 2021). Comparative studies are necessary to identify the safest and most effective NSC source for EV production, scaling, preservation, storage, mode of delivery, BBB crossing, targeted delivery, cellular uptake, and therapeutic potential for specific neurological conditions (Liu et al. 2023; Yamashita et al. 2018).

NSC‐derived EVs, even when produced from a similar cell source, represent a highly heterogeneous population with diverse molecular cargo, physical properties, and biological functions—posing significant challenges for their clinical standardization (Peruzzotti‐Jametti et al. 2021). This heterogeneity stems from variations in donor cell states, culture conditions, and EV biogenesis pathways, leading to inconsistent therapeutic outcomes and cargo profiles. Compounding the issue, there is currently a lack of standardized protocols for EV production, purification, characterization, quantification, and storage specific to NSC‐derived EVs (Li et al. 2023). Additionally, limited understanding of the mechanisms governing EV cargo sorting constrains the development of engineering strategies to selectively load therapeutic biomolecules into NSC‐EVs (Li et al. 2023; Yin et al. 2023).

To address these limitations, several promising approaches have emerged. First, subpopulation isolation techniques such as microfluidics, size‐exclusion chromatography, and immunoaffinity capture using markers like CD63 or NCAM can help obtain functionally uniform EV subsets for therapeutic use (Zhang, Huang, et al. 2025). Second, standardizing NSC culture conditions—including the use of 3D bioreactor systems, hypoxic environments, and serum‐free media—can reduce variability at the source and improve EV batch consistency (Rhim et al. 2023). Third, surface and cargo engineering strategies, such as incorporating targeting peptides (e.g., RVG, RGD) or utilizing controlled RNA/protein loading techniques like electroporation or light‐inducible dimerization, allow for precise customization of EV formulations (Nieland et al. 2023). Additionally, advanced single‐EV characterization tools—including nanoparticle tracking analysis, high‐resolution flow cytometry, and super‐resolution microscopy—enable quality assessment at the vesicle level and support reproducibility (Su et al. 2025). Finally, implementing GMP‐compliant pipelines that incorporate scalable isolation (e.g., tangential flow filtration) and robust quality control frameworks is essential for clinical‐grade production (Thakur and Rai 2024; Costa‐Ferro et al. 2024).

Collectively, these strategies provide a rational roadmap to overcome NSC‐EV heterogeneity and facilitate their safe, consistent, and effective application in CNS therapeutics.

Beyond the need for production and characterization standardization, optimization of storage and downstream handling is also critical for the clinical translation of NSC‐derived EVs. In relation to optimization of storage conditions, the International Society for Extracellular Vesicles (ISEV) recommends that EVs be conserved in isotonic buffers to prevent pH shifts during storage as well as during freezing and thawing procedures and stored at −80°C (Welsh et al. 2024). However, for therapeutic application and scale‐up production and distribution, perhaps lyophilization of EVs may improve their stability at higher temperatures (Yamashita et al. 2018). New methodologies have been developed to increase the capacity of isolated and quantified cell‐type‐specific EVs from body fluids based on the screening of specific protein surface markers derived from parental cells. For example, Ter‐Ovanesyan et al. (2024) developed efficient EV immuno‐isolation methods and applied them to isolate NRXN3+ EVs, specific neuron‐derived EVs, from CSF and plasma (Ter‐Ovanesyan et al. 2024). This new technique has been suggested as a universal methodology for the isolation of different cell‐type‐specific EVs (Ter‐Ovanesyan et al. 2024) such as NSC‐derived EVs. EV engineering strategies have emerged to optimize native EVs for improved targets, controlling release, and giving functional integration (Zhang, Wu, et al. 2024).

Treating CNS diseases with EVs is particularly challenging due to the presence of the BBB; therefore, various delivery methods are currently under investigation. The most effective approach for delivering NSC‐derived EVs across different neurological conditions needs to be optimized in a disease‐specific manner. Common delivery strategies include (a) intranasal administration, which allows rapid CNS absorption; (b) intravascular microbubbles combined with focused ultrasound to transiently open the BBB; (c) oral delivery of plant‐, milk‐, or bacteria‐derived EVs, which have been shown to reach the brain in some studies; (d) intravenous injection, though limited by short circulation time and rapid clearance; (e) intraperitoneal injection, which allows for high local uptake; and (f) subcutaneous injection, which shows minimal brain delivery. More invasive approaches include (g–h) intrathecal or intraventricular injection into the CSF, and (i) direct injection into specific brain regions or tumors (Nieland et al. 2023). Studies in mice have shown that intranasal administration of EVs is an effective and reliable method to bypass the BBB and deliver therapeutic agents to specific regions of the CNS (Nieland et al. 2023). In a clinical study, the nasal route was used for delivery of EVs derived from human umbilical cord blood MSCs for the treatment of ALS (NCT06D598202).

With advancements in EV isolation and enhanced cell specificity, EVs hold great promise for tissue‐specific applications by enabling targeted therapy delivery, improving treatment efficacy, and minimizing side effects (Zhang, Wu, et al. 2024). This can be achieved through surface modifications of EVs (e.g., conjugation with ligands or antibodies) to enhance tissue targeting, internal engineering (e.g., encapsulating therapeutic agents or genetic material) to modulate biological effects, and tuning of physical properties (e.g., size and charge) to optimize biodistribution, biodegradation, and cellular uptake (Zhang, Wu, et al. 2024). For instance, Alvarez‐Erviti et al. (2011) engineered “self” EVs derived from dendritic cells to express a neuron‐specific peptide (RVG) fused to the exosomal membrane protein Lamp2b, enabling targeted delivery to brain cells. These RVG‐targeted EVs effectively delivered siRNA to brain cells—including neurons, microglia, and oligodendrocytes—achieving targeted gene knockdown without eliciting immune responses or off‐target accumulation. This strategy was validated by a significant reduction in beta‐site APP‐cleaving enzyme 1 (BACE1) mRNA (60%) and protein (62%) levels in mice, highlighting its therapeutic potential for AD by targeting β‐amyloid peptide production (Alvarez‐Erviti et al. 2011). Overexpressing therapeutic molecules in parent cells is a straightforward strategy to enhance the therapeutic potential of EVs, as these molecules are subsequently enriched within the EVs and can exert stronger biological effects upon delivery to target cells (Geng et al. 2019; Yamashita et al. 2018). Accordingly, stroke rats treated intravenously with engineered EVs overexpressing miR‐126 showed improved functional recovery, enhanced neurogenesis, and reduced neuroinflammation, demonstrating the potential of miRNA‐enriched EVs in promoting post‐stroke neural repair (Geng et al. 2019). Another engineering approach that addresses the aforementioned challenges involves advanced surface modification techniques, including lipid insertion, chemical and enzymatic ligation, affinity binding, and metabolic labeling—each offering precise and customizable strategies to enhance EV targeting, stability, and therapeutic efficacy (Liu et al. 2023). Additionally, hybridization techniques enable the formation of nanovesicles that retain the surface properties of EVs while accommodating larger molecules (Louro et al. 2025). Advanced luminal loading techniques allow for the controlled incorporation of functional RNA and protein cargo into EVs, boosting their precision as CNS drug delivery platforms (Nieland et al. 2023). Finally, tissue engineering using biomaterials such as bioscaffolds, as discussed in Section 2.1, further enhances the potential for targeted and effective therapeutic applications.

Nevertheless, ethical and regulatory challenges remain significant considerations in the development of EV‐based therapies. The ISEV emphasizes that EV therapies are subject to regulations governing “tissues and cells” and “advanced therapy medicinal products” (ATMPs) (Lener et al. 2015). In the EU, tissue‐based products adhere to directives (2004/23/EC, 2006/17/EC) that focus on safety and quality standards, while ATMPs are subjected to stricter regulations due to manipulation and alterations in function. EVs, typically derived from human cells, may be classified under ATMP guidelines, necessitating adherence to good practice standards and thorough safety testing. In the U.S., EVs are not classified as human cell/tissue products (HCT/Ps), but safety concerns, including disease transmission, must still be addressed. Preclinical testing for EVs follows risk‐based approaches, like those for cell therapies. Adhering to these regulatory requirements ensures legal approval and fosters stakeholder trust, with future EV‐specific guidelines likely evolving from existing tissue and cell product regulations (Lener et al. 2015).

Altogether, despite the significant promise of NSC‐derived EVs for neurological therapy, several challenges must be addressed before their widespread clinical application. Key challenges include optimizing NSC sources, standardizing EV isolation and characterization, scaling up production while ensuring quality, and evaluating long‐term safety. Refining delivery strategies and advancing EV bioengineering, storage, and regulatory frameworks are essential for translating NSC‐EVs into clinically viable off‐the‐shelf therapies.

## 5 Conclusions

The CNS has limited regenerative capacity, with NSCs residing in neurogenic zones playing a role in self‐repair. While they hold promise for treating neurodegenerative and neuroinflammatory diseases, their endogenous repair capacity is often insufficient, particularly with aging, which compromises neurogenesis and contributes to disease progression. Dysfunction within the NSC niche, influenced by inflammation and environmental factors, further limits their regenerative potential. Approaches such as neurotrophic factors, gene therapy, and in vivo glial reprogramming show promise but face clinical challenges.

NSCs can be sourced from embryonic, fetal, or adult tissues, or reprogrammed from somatic cells. Despite promising preclinical and early clinical data in MS, ALS, and PD, challenges such as ethical concerns, tumorigenicity, and donor compatibility remain. NSC‐derived EVs, serving as a potent mechanism of action in NSC therapy, represent a promising cell‐free biotherapeutic approach due to their intrinsic neurogenic potential, offering advantages over cell therapy such as reduced immunogenicity and enhanced targeting capabilities. They provide neuroprotection, immunomodulation, and metabolic support, promoting neural regeneration in neurodegenerative and neuroinflammatory diseases.

Preclinical studies show that NSC‐EVs can mitigate neurodegeneration, reduce oxidative stress, and support mitochondrial function—modulating multiple pathways critical to CNS repair. Ongoing clinical trials underscore their potential as scalable, off‐the‐shelf therapeutics for stroke and neuroimmune disorders. However, translating NSC‐EVs into clinical therapies will require significant progress in optimizing delivery strategies, refining bioengineering approaches, and establishing comprehensive safety profiles. Key challenges include minimizing EV heterogeneity and off‐target effects, ensuring the long‐term safety of repeated administration, and eliminating unintended cargo such as oncogenic miRNAs or pro‐inflammatory cytokines. Clinical success will depend on a coordinated, multidisciplinary effort—integrating advanced bioengineering, standardized analytical methods, rigorous safety testing, and harmonized global regulatory frameworks. Central to this progress will be the development of GMP‐compliant manufacturing processes, validated potency assays, and clear regulatory pathways tailored to the unique complexity of NSC‐EV‐based therapeutics. Ultimately, bridging the fields of neural stem cell biology, nanotechnology, and clinical neuroscience will be crucial to unlock the full therapeutic potential of NSC‐EVs and bring transformative treatments to patients with currently untreatable neurological diseases.

## Author Contributions

**Eduardo H. Moretti, Ally L. Y. Lin** and **Sabah Mozafari:** writing – original draft; **Luca Peruzzotti‐Jametti, Stefano Pluchino, Sabah Mozafari:** writing – review and editing. **Stefano Pluchino** and **Sabah Mozafari:** conceptualization.

## Declaration of Generative AI and AI‐Assisted Technologies in the Writing Process

During the preparation of this work the authors used ChatGPT to check the grammar. After using this tool/service, the authors reviewed and edited the content as needed and took full responsibility.

## Conflicts of Interest

S.P. is founder, chief scientific officer, and shareholder (> 5%) of CITC Ltd. The other authors declare no conflicts of interest.

## Peer Review

The peer review history for this article is available at https://www.webofscience.com/api/gateway/wos/peer‐review/10.1111/jnc.70170.

## Data Availability

Data sharing is not applicable to this article because no new data were created or analyzed in this study.
